# Delayed Serotonin Syndrome Following the Concurrent Use of an Unregulated Supplement and Selective Serotonin Reuptake Inhibitors (SSRIs): A Case Report

**DOI:** 10.7759/cureus.93768

**Published:** 2025-10-03

**Authors:** Win Win Kyi, Elba B Peter, Keiko Carter, Joan O Otaru, Htar Htet Htet Wai

**Affiliations:** 1 General Medicine, Royal Gwent Hospital, Newport, GBR; 2 Medicine, Aneurin Bevan University Health Board, Newport, GBR; 3 Internal Medicine, University Hospital of Wales, Cardiff, GBR; 4 Geriatrics, Royal Gwent Hospital, Newport, GBR; 5 Acute Medicine, Lincoln County Hospital, Lincoln, GBR

**Keywords:** delayed onset of serotonin syndrome, depression, over-the-counter drugs, selective serotonin reuptake inhibitors, serotonin toxicity, unregulated supplements

## Abstract

Serotonin syndrome results from an excess accumulation of serotonin, which can be life-threatening if not promptly recognized. It typically arises from recent changes in antidepressant regimens or interactions with other medications and supplements. The diagnosis is clinical and features a spectrum of symptoms, from mild agitation and tremor to severe complications such as renal failure, seizures, and death. Early identification and intervention are crucial, typically resulting in the resolution of symptoms within 24 hours. With antidepressant use on the rise, serotonin syndrome is increasingly encountered in clinical practice. Patients prescribed serotonergic agents should be made aware of toxicity signs and cautioned about potential interactions with over-the-counter supplements.

This report describes a 27-year-old lady who presented with delayed onset of serotonin toxicity symptoms after two months of taking CutXtreme, an unregulated weight-loss supplement popularized on social media. She had a stable history on sertraline with no previous adverse effects. Discontinuation of both sertraline and CutXtreme, combined with supportive care, led to complete symptom resolution within 24 hours. This case emphasizes the importance of patient education on the potential risks of unregulated supplements, along with early recognition of serotonin toxicity and the need for clinical vigilance in unusual or atypical serotonin syndrome presentations.

## Introduction

Serotonin syndrome is a potentially life-threatening condition that results from excessive serotonergic activity in the central nervous system (CNS) [1,2]. It is commonly triggered by alterations in serotonergic medications, particularly when taken in combination with other pharmacologic agents [3]. Of particular concern is the interaction between selective serotonin reuptake inhibitors (SSRIs) and herbal products, as certain herbal components may potentiate serotonergic effects, increasing the risk of serotonin toxicity [4,5].

A triad of mental status changes, neuromuscular abnormalities, and autonomic instability characterizes the clinical presentation of serotonin syndrome [1,6]. Early symptoms typically develop within hours of changing medication regimens or initiating new pharmacological treatments [2]. Early detection and treatment are crucial, as timely intervention significantly improves prognosis. Most cases resolve within 24 to 48 hours after discontinuation of the offending agents and the initiation of supportive care [1,3].

To our knowledge, this is the first reported case of serotonin syndrome associated with CutXtreme, a commercially available thermogenic supplement widely marketed online [7]. It is also an unusual presentation as symptoms of serotonin toxicity emerged only after several weeks of supplement use, rather than shortly after initiation. The case highlights the risks of unregulated use of over-the-counter supplements in conjunction with prescribed serotonergic medications and the awareness of atypical delayed presentations of serotonin syndrome.

## Case presentation

A 27-year-old woman presented to the emergency department with the chief complaint, “My whole body feels weird, like vibrating, and I can’t stop shaking” for a week. She reported experiencing these symptoms for the past week, along with sneezing, nasal discharge, palpitations, and a tremor in her right hand. Her past medical history was significant for asthma and depression. She had no past surgical history and no known drug allergies. Her only regular long-term medication was sertraline 100 mg.

On examination, she appeared acutely distressed, agitated, and diaphoretic. She was afebrile, and her vital signs were within normal limits. There was no evidence of meningismus. She was alert and oriented to time, place, and person. Neurological examination revealed bilateral lower limb tremors, inducible myoclonus in the lower extremities, and hyperreflexia with brisk reflexes in all four limbs. Cranial nerve and sensory examinations were normal. Pupils were dilated (mydriasis) but reactive to light. Cardiopulmonary, abdominal, and dermatological examinations were unremarkable.

All laboratory and diagnostic studies, including a complete blood count, basic metabolic panel, liver function tests, electrolyte panel, and renal function tests, were within normal limits. Thyroid function tests and creatine kinase levels were also within normal ranges (Table 1). An electrocardiogram (ECG) revealed sinus tachycardia, with no other abnormalities noted.

**Table 1:** Serial Laboratory Investigations Comparison of admission (13 June) and pre-discharge (15 June) laboratory values with corresponding reference ranges.

Laboratory test	13 June (on admission)	15 June (before discharge)	Reference range
White blood cell (WBC) count	10.7 × 10⁹/L	8.2 × 10⁹/L	4.0-11.0 × 10⁹/L
Hemoglobin (Hb)	140 g/L	120 g/L	115-165 g/L
Platelet (PLT) count	494 × 10⁹/L	386 × 10⁹/L	150-400 × 10⁹/L
Calcium (adjusted)	2.63 mmol/L	2.33 mmol/L	2.20-2.60 mmol/L
Protein	80 g/L	61 g/L	60-80 g/L
Phosphate	0.93 mmol/L	1.25 mmol/L	0.80-1.50 mmol/L
Alkaline phosphatase	98 U/L	76 U/L	30-130 U/L
C-reactive protein (CRP)	<10 mg/L	<10 mg/L	<10 mg/L
Creatine kinase	82 U/L	58 U/L	25-200 U/L
Alanine transaminase	25 U/L	23 U/L	<41 U/L
Sodium	141 mmol/L	139 mmol/L	133-146 mmol/L
Potassium	4.0 mmol/L	4.1 mmol/L	3.5-5.3 mmol/L
Urea	3.9 mmol/L	3.0 mmol/L	2.5-7.8 mmol/L
Creatinine	82 µmol/L	61 µmol/L	46-92 µmol/L

The patient's presentation was clinically consistent with a mild form of serotonin syndrome. Differential diagnoses, including meningitis, thyrotoxicosis, malignant hyperthermia, and neuroleptic malignant syndrome, were taken into consideration but were ruled out based on the patient's symptoms and test results. Serotonin syndrome is rarely associated with SSRI monotherapy. Additional questioning revealed concurrent use of a thermogenic supplement, CutXtreme, for approximately two months, which raised concerns about a possible interaction. Upon further detail, only one tablet of CutXtreme was taken since initiation, and there were no other specific changes in the dosage of either sertraline or CutXtreme prior to the onset of symptoms.

The diagnosis of serotonin syndrome was established clinically, despite the atypically delayed presentation. Both sertraline and CutXtreme were discontinued immediately. The patient was managed with supportive care, including symptomatic treatment with lorazepam administered as needed for agitation. She remained hemodynamically stable throughout her 24-hour observation period, during which her symptoms gradually improved. Following resolution of her symptoms, she was discharged with appropriate counseling on the recognition and prevention of serotonin syndrome and advised to avoid unregulated supplements in the future.

## Discussion

SSRIs are widely prescribed medications for the treatment of depression, anxiety, panic disorder, eating disorders, and obsessive-compulsive disorder [1,7]. Serotonin, or 5-hydroxytryptamine (5-HT), is a neurotransmitter found in the central and peripheral nervous system. Excessive serotonin is taken back by the active transport mechanism in presynaptic neurons or locally metabolized by monoamine oxidase. Systemic metabolism is facilitated through cytochrome P-450 mixed-function oxidase (MFO) microsomal enzymes [8].

A recently increased dose of a chronic medication, or a new addition to an extensive medication regimen, is an essential component of the history that may provide the diagnosis [2]. Use of over-the-counter medications or dietary supplements in addition to prescribed serotonergic medications is also an important etiology [4].

The majority of cases of serotonin syndrome present within 24 hours, and most within six hours, of a change in dose or initiation of a drug [2]. In contrast, the patient in this case presented atypically, with symptoms emerging only after several weeks of CutXtreme use-well beyond the typical onset window for serotonin syndrome. CutXtreme is a widely marketed thermogenic supplement, particularly popular on social media platforms such as TikTok. It is promoted as a product designed to support weight management, enhance energy and focus, and improve mental clarity. Currently, no published data describe pharmacodynamic or pharmacokinetic interactions between sertraline and CutXtreme. As such, the possibility of a delayed or cumulative interaction effect remains speculative.

CutXtreme remains unclear whether the formulation possesses serotonergic or other pharmacologically active properties that may interact with sertraline. The listed ingredients include L-tyrosine, caffeine, N-acetyl-L-carnitine, vitamin B5, L-phenylalanine, vitamin B12, biotin, chromium, and various plant extracts. However, based on current knowledge, there is no established evidence of interaction between sertraline and these individual components that would precipitate serotonin syndrome [7].

CutXtreme contains a combination of ingredients that, when combined with sertraline, may pose an interaction risk or influence serotonergic effects [7]. Key elements include L-tyrosine and L-phenylalanine, which do not directly increase serotonin levels; however, their effect on catecholamines may contribute to CNS stimulation, which may amplify some sertraline side effects such as anxiety, jitteriness, insomnia, or increased heart rate [9]. Green tea extract and guarana extract (which contains caffeine) are stimulants that might boost CNS activity and perhaps intensify adverse effects [10]. Bitter orange peel contains synephrine, a stimulant that can raise heart rate and blood pressure [11]. N-acetyl-L-carnitine is considered safe; however, its effects on serotonin are unclear [7].

None of these are classical serotonergic agents like 5-HTP, but stimulant and neurotransmitter precursor effects could contribute to serotonergic overload or adverse reactions when combined with sertraline, especially in the context of unregulated supplements with variable dosages. Because the supplement is unregulated, there may also be undisclosed ingredients with serotonergic activity that increase the risk of serotonin syndrome when combined with SSRIs like sertraline [7].

One of its components, caffeine, is primarily metabolized by the liver enzyme CYP1A2, which also contributes to the metabolism of certain antidepressants. However, most newer antidepressants, particularly SSRIs such as sertraline, are minimally affected by this metabolic pathway. Although concurrent use of caffeine and serotonergic agents may exacerbate symptoms of anxiety and depression, current literature does not provide evidence that caffeine contributes to the development of serotonin syndrome when used concomitantly with SSRIs [12].

The diagnosis of serotonin syndrome is made solely on clinical grounds. Therefore, a detailed history and thorough physical and neurologic examinations are essential [2]. The Hunter Criteria are the most accurate diagnostic criteria for serotonin syndrome, with an 84 percent sensitivity and a 97 percent specificity (Table 2) [6].

**Table 2:** Hunter Serotonin Toxicity Criteria Diagnosis of serotonin syndrome is supported when a patient with known serotonergic agent exposure meets any one of these criteria. The syndrome is typically not a diagnosis of exclusion when the Hunter criteria are satisfied [6].

Requirement	Clinical findings (one of the following)
Serotonergic agent exposure	AND any one of the following conditions:
	Spontaneous clonus
	Inducible clonus plus agitation or diaphoresis
	Ocular clonus plus agitation or diaphoresis
	Tremor plus hyperreflexia
	Hypertonia plus temperature > 38°C plus ocular clonus or inducible clonus

The patient’s presentation satisfied the Hunter serotonin toxicity criteria with the clinical manifestation of inducible myoclonus, agitation, diaphoresis, tremor, and hyperreflexia [6]. Serotonin syndrome encompasses a spectrum of diseases in which the intensity of clinical symptoms appears to coincide with the level of serotonergic activity [2]. Once serotonin syndrome has been identified and the offending agent has been removed, supportive care is the mainstay of treatment [1,2]. In this patient, both sertraline and CutXtreme were stopped, and her symptoms resolved within 24 hours [2].

## Conclusions

Antidepressant prescriptions have increased globally due to increasingly stressful lifestyles. Of which, SSRIs are the most prescribed medication in primary and psychiatric settings. While serotonin syndrome typically presents shortly after the initiation or dose adjustment of serotonergic agents, clinicians should remain vigilant for delayed onset, as rare cases of late presentation have been reported.

It is essential to carefully counsel all patients who initiate mediation to be aware of serotonin toxicity and syndrome and the importance of reporting symptoms. Patients should be counseled about potential interactions with any medications they take, including over-the-counter medications and herbal supplements. In the absence of precise evidence, caution should be exercised when using CutXtreme in combination with sertraline. Given the widespread use of over-the-counter supplements, enhanced regulation and quality assurance are crucial to safeguarding patient safety and reducing the risk of adverse interactions with serotonergic medications such as SSRIs. Patients should be advised to consult with their healthcare providers before changing any medications or adding supplements.
